# Testing Parenting Self-Esteem as an Indicator of Mothers and Fathers Who Are at Risk for Aversive Responses to Disruptive Child Behavior

**DOI:** 10.1007/s10802-024-01231-8

**Published:** 2024-08-09

**Authors:** Sierra R. Hightower-Henson, Brian T. Wymbs

**Affiliations:** Department of Psychology, Ohio University, Athens, OH USA

**Keywords:** Parenting behaviors, Disruptive child behaviors, Parent efficacy, Parent satisfaction, Moderation

## Abstract

Extensive literature demonstrates that parents of children with disruptive behaviors consistently report lower parenting self-esteem (i.e., satisfaction and efficacy) compared to parents of children without disruptive behaviors. However, little is known about whether having low parenting self-esteem results in negative parenting behavior while managing disruptive child behavior, and whether associations vary in strength depending on the clinical significance of the child’s disruptive behavior. The current study examines 90 parent couples who were randomly assigned to interact with a 9- to 12-year-old confederate exhibiting either typical or disruptive behaviors. Parenting self-esteem moderated the association between disruptive child behavior and positive parenting behavior, such that mothers with low efficacy had a stronger positive association between disruptive child behavior and positive parenting behaviors. However, fathers with low efficacy had a stronger negative association between disruptive behaviors and positive parenting behavior. Exploratory analyses yielded mixed results. Specifically, mothers with low self-esteem and a child with ADHD had a stronger negative association between disruptive child behaviors and positive parenting compared to mothers who interacted with a confederate or did not have a child with ADHD. Results from the current study extend findings regarding the influence of parenting self-esteem on the association of disruptive child behavior and parenting behaviors, as moderating effects of parenting self-esteem was demonstrated for both mothers and fathers within the study.

Parenting is an inherently demanding and stressful role, but this role is even more challenging for parents raising children who exhibit chronic disruptive behaviors. Disruptive child behaviors can be characterized by inattention, defiance, interrupting others, and tantrum-like behavior, which are associated with disorders such as attention-deficit/hyperactivity disorder (ADHD) and oppositional defiant disorder (ODD; American Psychiatric Association, 2013). Research demonstrates that disruptive child behaviors are driving forces of parenting difficulties (Barkley, 1981; Pelham & Lang, 1999; Wymbs & Pelham, 2010). Given the use of aversive parenting strategies (e.g., rejection, hostility, coldness) exacerbates risk of child disruptive behaviors (Johnston & Mash, 2001) and perpetuates the cycle of distress in these families (Patterson, 1982), identifying variables that attenuate the association between disruptive child behaviors and aversive parenting behaviors (i.e., more negative and less positive parenting) in moments of distress may lead to strategies to disrupt coercive cycles.

Although we have known for some time that children behave in ways that elicit adverse reactions from parents (Bell & Chapman, 1986), it is also clear that not all parents are provoked to more negative and less positive parenting in response to managing disruptive child behavior. In fact, it may only be parents with certain risk factors who are more likely to respond aversively in the moment to disruptive child behavior (Johnston & Chronis-Tuscano, 2015). For the purpose of ultimately delivering needed assistance to those parents who are at greatest risk of being triggered by disruptive child behavior, this study set out to test whether one potential risk variable (parenting self-esteem) moderates the association between disruptive child behaviors and aversive parent responses.

## Disruptive Child Behavior and Parenting Behavior

Across correlational and experimental studies, research has demonstrated that disruptive child behavior is associated with, or elicits, aversive parenting behavior (i.e., more negative and less positive parenting). For example, in samples of youth with and without ADHD, increases in disruptive child behaviors predict increases in negative parenting behaviors, such as hostility, coldness, and engaged in more coercive parenting over time (e.g., Breaux & Harvey, 2019; Pagani & Fitzpatrick, 2018) as well as decreased positive parenting behaviors (i.e., warmth, consistency; Demmer et al., 2018). Additionally, experimental manipulations of child behavior via medication manipulations (Barkley et al., 1984) and child confederates (Pelham et al., 1998) have found that children behaving disruptively elicited more negative and less positive parenting behaviors in the moment than children behaving typical/non-disruptively.

The next step for this line of research is to highlight parents who are especially prone to parenting aversively in response to disruptive child behavior. After all, parents are not equally likely to react negatively to children behaving inappropriately. Indeed, there are many parents who effectively manage behaviors in difficult circumstances (for review, see Fang et al., 2021). Identifying factors associated with maladaptive parenting in response to disruptive child behavior may be an important development for at-risk parents raising children with chronic and pervasive disruptive behavior problems, such as those with ADHD. For example, parents of children with ADHD who endorse high levels of risk factors associated with aversive responding could especially benefit from evidence-based parenting interventions. One such risk factor may be low parenting self-esteem.

## Parenting Self-Esteem as a Potential Risk Factor

Self-efficacy theory developed by Bandura (1977) posits that a person’s ability to attain the desired outcome through their ability and actions will motivate the individual and their effort, as well as instill persistence. This theory states that people develop their personal sense of efficacy through experiences, including “mastery experiences.” A mastery experience is the most influential in developing efficacy because it provides evidence that an individual can succeed in the situation they are in, and this success contributes to one’s personal efficacy. However, if success builds this area, then a failure in mastery experience also undermines a person’s sense of efficacy (Bandura, 1997). Negative experiences influence a person’s perception of their abilities, such that a parent with a child who exhibits chronic disruptive behaviors may not believe that they are capable of parenting after having to redirect their child repeatedly. These negative experiences may also take a negative toll on a parent’s mental, physical, and emotional well-being. Thus, parents may not feel equipped to handle challenging encounters with their child. As such, the unsuccessful experiences of some parents managing youth disruptive behavior may contribute to their feelings of low sense of efficacy as a parent, and it may be those with lower efficacy who are especially at risk for engaging in aversive parenting behaviors in response to managing future disruptive child behaviors.

Parenting self-esteem is consistent with the bi-dimensional view of self-esteem (i.e., satisfaction and efficacy). The first construct of satisfaction is focused on the feelings of contentment and like of their role as a parent (Bandura, 1977; Teti & Gelfand, 1991). The second construct of efficacy focuses on problem-solving abilities as a parent, capability within their role, and how competent and confident that parent feels in responding to their child’s behaviors (Bandura, 1977; Teti & Gelfand, 1991). Both constructs are important in defining parental self-esteem in that they define a personal evaluation of their parenting role.

Prior investigations of associations between constructs of parenting satisfaction and efficacy and disruptive child behaviors have found that low levels of parenting satisfaction and efficacy were found to be negatively associated with disruptive child behaviors (Johnston, 1996; Johnston & Mash, 1989; Ohan et al., 2000). Specifically, mothers and fathers who felt less satisfied or efficacious as a parent tended to report that their children had more disruptive behavior. Extending this work with typical, community samples to a clinic-referred sample, Mash and Johnston (1996) found that the negative association between parents’ ratings of parenting self-esteem and their child’s reported disruptive behaviors were stronger among the parents of children with ADHD than of parents of children without ADHD. Additionally, Johnston (1996) found that parents who had children with ADHD and high levels of oppositional behaviors reported the lowest levels of parenting self-esteem compared to parents of children with only a diagnosis of ADHD and parents of children with ADHD and low levels of oppositional behaviors. Thus, elevated disruptive child behaviors appear to be associated with lower parent-reported levels of parenting satisfaction and efficacy within clinical and non-clinical samples.

Significant associations are also evident between parent satisfaction and efficacy and self-reported use of negative parenting behavior (Johnston, 1996; Ohan et al., 2000). A study done by Ohan et al. (2000) highlighted significant positive correlations between parenting satisfaction and efficacy and more positive, easy-going, and low-conflict parenting behaviors in parents. Similarly, a study done by Johnston and Patenaude (1994), showed that parents who had lower ratings of parent self-esteem reported more negative parenting responses to the disruptive behaviors of their child than parents with higher parenting satisfaction and efficacy. Previous research has also highlighted a negative association between negative parenting behaviors and parenting self-esteem, such that parenting self-esteem tends to improve after receiving behavioral parent training and other interventions that successfully reduce negative parenting behaviors (Pisterman et al., 1992). Additionally, parents of children with ADHD and high oppositional behavior report engaging in more aversive parenting behaviors compared to parents of children with ADHD and parents of ADHD and low oppositional behaviors (Johnston & Patenaude, 1994). Altogether, links between low parenting satisfaction and efficacy and low positive and high negative parenting behaviors appear robust within clinical (Johnston & Patenaude, 1994; Pisterman et al., 1992) and non-clinical samples (Ohan et al., 2000).

Nonetheless, the evidence underscoring links between parenting self-esteem, disruptive child behavior, and aversive parenting is limited in several keyways. First, prior research has only examined correlations between reports of parenting self-esteem, disruptive child behavior, and parenting behavior averaged over time, which likely misses nuances of parent reactions to moments of disruptive child behavior. Observing parents’ immediate reactions to overt disruptive behaviors is necessary to determine whether any potential risk factors, such as low parenting satisfaction and low parenting efficacy, indeed identify parents especially prone to aversive reactions in relevant, common moments of parenting. It remains unclear whether the association between disruptive child behavior and ineffective parenting in response may be stronger or weaker for parents with higher or lower parenting satisfaction and efficacy. Second, studies have only evaluated links between parenting self-esteem, disruptive child behavior, and parenting behaviors with mother–child dyads or through self-report of disruptive child behaviors and parenting behaviors, which misses the nuance of interactions in father-child dyads or observations of parent–child interactions. The current study was designed to address the current gaps in the literature by examining parenting self-esteem as a possible indicator of parents who are prone to aversive parenting behavior assessed via partner- and observer-report during moments of managing disruptive child behavior. It is beneficial to understand how feelings of satisfaction and efficacy moderate the link between disruptive child behaviors and aversive parenting for mothers and for fathers. If moderation is demonstrated, this may signal a need to target parent couples including one or two partners with lower parenting satisfaction and efficacy for parenting interventions designed to address how they respond to disruptive child behaviors.

## Current Study

The primary aim of the current study is to investigate parenting satisfaction and efficacy as moderators of causal links between disruptive child behavior and aversive parenting behaviors. As part of a larger study (Wymbs & Pelham, 2010), 90 mother-father couples were randomly assigned to interact briefly with a “disruptive” child confederate exhibiting ADHD/ODD-like behavior or with a “typical” confederate exhibiting developmentally-appropriate behavior. Relying on confederate behavior as the child behavior main effect allowed for us to test directly how parents respond in-the-moment to ADHD/ODD-like or typical child behavior. Indeed, Wymbs (2011) found that parents managed “disruptive” confederates in this sample more negatively and less positively than “typical” confederates. Before the child interactions, both parents reported on their own parenting self-esteem and immediately afterward, they reported on the quality of their partner’s parenting during the interaction. Trained observers also coded parenting behavior. We hypothesized that the positive association between disruptive confederate behavior and negative parenting behaviors will be stronger in parents who report low satisfaction or low efficacy relative to parents with higher levels of parenting satisfaction and efficacy. We also hypothesized that the negative association between disruptive confederate behavior and positive parenting behavior will be stronger in parents who report low satisfaction or low efficacy relative to parents with higher levels of parenting satisfaction and efficacy.

Additionally, prior research indicates that associations between parenting self-esteem, “disruptive” behavior, and parenting behavior occasionally vary depending on the ADHD/ODD status of parents' own children (Johnston & Patenaude, 1994; Pisterman et al., 1992; Wymbs & Pelham, 2010). Thus, there is a reason to explore this as an additional moderator. To this end, this study explored whether parenting satisfaction and efficacy strengthened or weakened the associations between disruptive confederate behavior and parenting behavior differentially by ADHD/ODD status of the parent couples’ own child.

## Method

### Participants

The current study draws data from a previous study done by Wymbs & Pelham (2010), which was designed to investigate the causal effects of disruptive child behaviors on interparental communication among 90 parent couples. Participants were recruited through newspaper and radio advertisements, information posted in physician’s offices, and flyers distributed through local elementary schools. Couples were required to have lived together for two years and both were active parents with their child at home. Further, recruitment was designed to over-sample parent couples who had children with ADHD and/or ODD diagnoses (n = 51) as well as recruit parent couples who had children without ADHD or ODD histories (n = 39).

Table 1 highlights the demographic similarities (i.e., age, race/ethnicity, marital status, household income) between the parent couples of children with ADHD and parent couples of children without ADHD. There was only one of 12 variables that differed significantly between the groups, which was the experience of the parent. Parents of children without ADHD reported greater parenting experience compared to parents of children with ADHD. There was also a statistical trend for mothers of children without ADHD to report having a higher level of education compared to mothers of children with ADHD. These factors have previously been controlled for within studies using this data set and were controlled for in the current study as well to eliminate potential statistical confounds.

**Table 1:** Sociodemographic characteristics of mothers of children with and without ADHD

	**All Mothers**	**ADHD** (*n* = 51)^a^ M *(SD) or %*	**Non-ADHD** (*n* = 39)^a^ M *(SD) or %*	***p***
Age	39.68 *(6.23)*	39.41 (*6.15*)	40.03 (*6.39*)	0.65
Relationship Status				0.28
Married	94.80%	92.16%	97.44%	
Single Living with Partner	5.20%	7.84%	2.56%	
Highest level of education^b^	6.70 *(1.66)*	6.41 *(1.80)*	7.08 (*1.38)*	0.06
Race/Ethnicity				0.46
White	97.80%	96.00%	100.00%	
Black or African American	1.1%	2.00%	0.00%	
Hispanic, Latino, Spanish American	0.00%	0.00%	0.00%	
Asian American or Pacific Islander	0.00%	0.00%	0.00%	
Native American	0.00%	0.00%	0.00%	
Mixed Race	1.1%	2.00%	0.00%	
PSOC Satisfaction	39.46 *(6.75)*	37.16 *(6.73)*	42.46 *(5.53)*	<.01
PSOC Efficacy	29.03 *(4.91)*	27.49 *(5.31)*	31.05 *(3.46)*	<.01
OC Neg. Parenting	0.22 *(0.34)*	0.23 *(0.38)*	0.20 *(0.29)*	.437
OC Pos. Parenting	110.50 *(51.63)*	113.94 *(56.29)*	106.00 *(45.13)*	.362
PR. Neg. Parenting	1.88 *(1.42)*	1.98 *(1.58)*	1.74 *(1.19)*	.436
PR. Pos. Parenting	8.58 *(1.56)*	8.37 *(1.65)*	8.85 *(1.41)*	.154

	**All Fathers**	**ADHD** (*n* = 51)^a^ M *(SD) or %*	**Non-ADHD** (*n* = 39)^a^ M *(SD) or %*	***p***
Age	41.96 *(6.75)*	41.92 (*6.72*)	42.00 (*6.87*)	0.96
Relationship Status				0.28
Married	94.80%	92.16%	97.44%	
Single Living with Partner	5.20%	7.84%	2.56%	
Highest level of education^b^	6.48 *(1.96)*	6.22 *(2.02)*	6.82 *(1.85)*	0.15
Race/Ethnicity				0.59
White	92.20%	94.10%	89.60%	
Black or African American	2.20%	2.00%	2.60%	
Hispanic, Latino, Spanish American	1.10%	0.00%	2.60%	
Asian American or Pacific Islander	1.10%	0.00%	2.60%	
Native American	0.00%	0.00%	0.00%	
Mixed Race	3.30%	3.90%	2.60%	
PSOC Satisfaction	37.76 *(7.23)*	34.51 *(6.86)*	42.00 *(5.29)*	<.01
PSOC Efficacy	29.07 *(4.87)*	27.65 *(5.18)*	30.92 *(3.74)*	<.01
OC Neg. Parenting	0.19 *(0.32)*	0.22 *(0.35)*	0.14 *(0.26)*	.20
OC Pos. Parenting	85.81 *(40.47)*	84.80 *(43.14)*	87.13 *(37.19)*	.79
PR. Neg. Parenting	1.68 *(1.17)*	1.88 *(1.35)*	1.41 *(.82)*	.06
PR. Pos. Parenting	8.81 *(1.51)*	8.14 *(1.80)*	9.23 *(.84)*	.02

A log transformation was conducted on OC, Neg. Parenting

*ADHD* attention-deficit/hyperactivity disorder, *PSOC* Parenting Sense of Competence Scale, *PR* Partner ratings of parenting behavior, *OC* Observer-coded parenting behavior, *Neg.* Negative, *Pos.* Positive

^a^Number of mothers or fathers

^b^Response scale for level of education ranged from 1 to 9, with the following rating choices: 1 = less than a seventh-grade education; 2 = junior high school (9th grade); 3 = partial high school; 4 = high school graduate or GED; 5 = specialized training; 6 = partial college; 7 = associate’s or two-year degree; 8 = standard college or university education; 9 = graduate professional training

### Procedure

All study procedures were approved by the University of Buffalo Institutional Review Board and these procedures are thoroughly described elsewhere (Wymbs & Pelham, 2010). After parent couples provided informed consent and confirmed their demographic information, they were provided information on the triadic interaction task sequence. Couples were informed that they would be interacting with an unfamiliar child who had similar characteristics (i.e., sex, race, age) to their own child. The parent couples were encouraged to interact naturally with the child during this interaction. Couples were reminded that the purpose of the study was to learn more about the process of co-parenting, which meant that they were asked to problem solve with one another to address child behavior management issues as needed. Unbeknownst to the couples, the child within these interactions was a confederate (see Child Behavior section below for specific confederate procedures).

The triadic interaction began with *a cooperative task* (8 min), which involved having the parent couples engage with the child by helping them build the tallest Jenga block tower within the shortest amount of time and to help rebuild the tower if it fell so they could play again. The couples then engaged in a *parallel task* (7 min) with the child that included each parent balancing a checkbook and ensuring that the child completed a math worksheet. During the *free play task* (7 min), couples were told to engage in play with the child by utilizing the toys that were available in the room and allow for the child to direct the play. Finally, the *clean-up task* (3 min) required parent couples to ask the child to clean up the toys without their help. With the exception of the cooperative task, which utilized Jenga instead of an Etch-a-Sketch to allow for triadic (parent-parent-confederate child) interactions, the remaining tasks were the same as those used in Pelham and Lang studies (Lang et al., 1999; Pelham et al., 1997, 1998). Researchers have also utilized these tasks to assess factors that impact the parent–child relationship quality (Johnston et al., 2002). Upon collecting all data following the experimental manipulation, parent couples were debriefed on the purpose of the study, including the fact that children were confederates.

### Measures

#### Predictor

##### Child Behavior

Seven 9- to 12-year-old children (five boys and two girls) went through extensive training to enact two scripted behavioral roles: one that dictated they behave like developmentally-appropriate disruptive children with ADHD and ODD (i.e., “Disruptive” confederates) and the other script requiring the children behave like developmentally-appropriate typical children without disruptive behaviors (i.e., “Typical” confederates). All confederates were trained to enact both typical and disruptive roles, but they were randomly assigned to enact only one role with each parent couple for the duration of the child interaction. Reliability checks were conducted during every interaction to assure the integrity of the confederate behavior manipulation. Trained observers utilized checklists to track the behavior of the confederates and recorded whether specific behaviors were exhibited or omitted correctly according to the scripts for each role. Behavior tracking confirmed the integrity of the typical (*M* = 93%, *SD* = 5%, range = 82–99%) and disruptive (*M* = 88%, *SD* = 4%, range = 78–98%) confederate roles. Altogether, 47 couples were randomly assigned to interact with a disruptive confederate and 43 were assigned to interact with a typical confederate. When considering the ADHD/ODD status of the parent couples’ own child, assignment was as follows: 1) 27 couples with children with ADHD/ODD were randomly assigned to interact with a “disruptive” confederate; 2) 24 couples with children with ADHD/ODD were randomly assigned to interact with a “typical” confederate; 3) 20 couples with children without ADHD/ODD were randomly assigned to interact with a “disruptive” confederate, and 4) 19 couples with children without ADHD/ODD were randomly assigned to interact with a “typical” confederate. Confederate child behavior was coded dichotomously with 1 = “disruptive behavior” and 0 = “typical behavior”.

#### Outcomes

##### Observer-Coded Parenting Behavior

Parenting behaviors were coded using the Family Observation Schedule (FOS; Dadds et al., 1987), which is a frequency-based coding system that captures parenting behaviors during interactions with disruptive children (Dadds et al., 1987) Undergraduate assistants coded video recordings of the interactions and noted parents’ use of positive (e.g., questions, praise, social attention) and negative (e.g., negative feedback, threat of punishment, criticism) parenting behaviors directed toward the child. Total frequency of positive parenting was a dependent measure. Due to the positive skew observed in negative parenting, a log transformation was performed to increase the validity of the variable. Reliability for positive (α = 0.98) and aversive (α = 0.98) parenting were acceptable (Wymbs, 2011).

##### Partner-Reported Parenting

After the interaction, parents completed a measure that was adapted from Fincham and Linfield’s (1997) Positive and Negative Quality in Marriage Scale. The scale asked the parent to rate a single item on how positive (ranging from 1 = “not at all positive” to 10 = “completely positive”) and negative (ranging from 1 = “not at all negative” to 10 = “completely negative”) they perceived their partner’s parenting of the child be during the child interaction.

#### Moderators

##### Primary Moderator

Prior to the triadic interaction, each parent self-reported their parenting self-esteem on the Parenting Sense of Competence Scale (PSOC; Johnston & Mash, 1989). The PSOC is a 16-item self-report measure that has parents rate each item on a 6-point Likert scale that has two subscales: satisfaction and efficacy. The satisfaction subscale includes 9-items and scores are calculated by reverse scoring and then summing the items. The efficacy scale is 7-items, and the efficacy score is calculated by summing the items. For these subscales, high scores indicate greater believed efficacy and satisfaction within their parental role. Ohan et al. (2000) found high internal consistency for both the satisfaction (for mothers and fathers, Cronbach’s α = 0.80) and efficacy (for mothers, Cronbach’s α = 0.80 and for fathers, Cronbach’s α = 0.77) subscales. In the current study, the internal consistency was fair for both satisfaction (for mothers, Cronbach’s α = 0.81 and for fathers, Cronbach’s α = 0.80) and efficacy (for mothers, Cronbach’s α = 0.74 and for fathers, Cronbach’s α = 0.77).

##### Exploratory Moderators

ADHD/ODD status of the parent couples’ own child was tested as a moderator alongside parent self-esteem (i.e., satisfaction and efficacy). The presence of ADHD was assessed by the researchers using evidence-based practices and guidelines (Pelham et al., 2005). Specifically, one parent from each couple completed standardized rating scales assessing whether their child exhibited clinically significant ADHD or ODD symptoms (Disruptive Behavior Disorder Rating Scale; Pelham et al., 1992) and functional impairment (Impairment Rating Scale; Fabiano et al., 2006). The child’s teacher was also asked to complete the same rating scales. Children were diagnosed with ADHD if they met the criteria for ADHD from the *Diagnostic and Statistical Manual of Mental Disorders* (4th edition; *DSM-IV*; American Psychiatric Association, 1994). The criteria for ADHD included (a) exhibiting six or more symptoms of inattention, six or more symptoms of hyperactivity-impulsivity, or both, according to parent and teacher reports; (b) demonstrating clinically significant symptoms and impairment in home and school settings, and (c) showing symptoms and impairment across home and school settings prior to the age of 7 years-old. As indicated above, 51 couples had children meeting criteria for ADHD, and 39 couples had a child who did not meet criteria for ADHD. Twenty-two children (43.1%) with ADHD also met *DSM–IV* criteria for ODD. Child ADHD/ODD status was coded as 1 = “child with ADHD/ODD” and 0 = “child without ADHD/ODD”.

### Analytic Approach

All analyses were conducted using IBM Statistical Package for the Social Sciences (SPSS) version 28 and were conducted separately for mothers and fathers. To assess for outliers within the data set, descriptive statistics (i.e., mean and standard deviations) were computed for all measures, and correlations were conducted between confederate child behavior, child ADHD/ODD status, satisfaction, efficacy, and parenting behaviors (partner- and observer-rated). To assess the moderating effects of parent satisfaction and efficacy on the association between child behavior and parenting behavior, linear regression analyses were used. In each linear regression analysis, all predictors were centered, and length of relationship and parent education served as covariates for all analyses and were entered into each model. In the absence of significant interactions, main effects were not interpreted as these findings have been presented elsewhere (Wymbs, 2011). Exploratory three-way interactions examining the moderating effects of parenting self-esteem (i.e., satisfaction and efficacy) and child ADHD/ODD status on parenting behavior were examined using linear regression analyses. The PROCESS procedure for SPSS Version 4.1 (Hayes, 2013) was utilized for all moderation analyses, and in this procedure, all predictors were mean-centered, and length of relationship and parent education were covariates for all analyses. Pearson Product-Moment correlations were conducted separately for mothers and fathers are presented in Table 2.

**Table 2:** Correlations for predictor, moderators, and parenting outcomes among mothers and fathers

Variable	1	2	3	4	5	6	7	8
1. Confed Stat	—	-0.10	-0.06	0.02	-0.42^**^	0.44^**^	0.31^**^	0.52^**^
2. PSOC Sat	-0.10	—	0.48^**^	-0.52^**^	0.39^**^	-0.23^*^	0.20	-0.02
3. PSOC Eff	0.08	0.39^**^	—	-0.34^**^	0.31^**^	-0.28^**^	0.25^*^	-0.08
4. Child ADHD Status	0.02	-0.39^**^	-0.36^**^	—	− 0.25^*^	0.20	-0.03	0.13
5. PR Pos. Parenting	-0.55^**^	0.09	0.11	−0 .15	—	− 0.80^**^	0.03	-0.27^**^
6. PR Neg. Parenting	0.41^**^	-0.03	-0.04	− 0.08	− 0.65^**^	—	0.03^*^	0.40^**^
7. OC Pos. Parenting	0.35^**^	-0.22^*^	-0.08	0.08	− 0.22^*^	0.26^*^	—	0.31^**^
8. OC Neg. Parenting	0.61^**^	-0.11	0.06	-0.04	-0.32^**^	0.28^**^	0.44^**^	—

*N* = 90. Mothers’ correlations shown in the bottom left, fathers’ correlations shown in the top right

A log transformation was conducted on OC Neg. Parenting

Higher scores on the efficacy and satisfaction subscales indicate greater feelings of satisfaction and efficacy, respectively

Confederate status (1 = Disruptive and 0 = Typical). Child ADHD Status (1 = Child with ADHD and 0 = Child without ADHD). Partner-reported parenting was rated on a 10-point Likert scale (1 = not at all to 10 = completely)

*PR* Partner ratings of parenting behavior, *OC* Observer coded parenting behavior, *PSOC* Parenting Sense of Competence Scale, *Sat.* Satisfaction, *Eff.* Efficacy

^*^p < 0.05; ^**^p < 0.01

## Results

### Preliminary Analyses

Pearson Product-Moment correlations were run separately for mothers and fathers are presented in Table 2. Correlations demonstrated that confederate child behavior was associated with observer and partner-reported positive and negative parenting for both mothers and fathers, which is consistent with previous reports indicating that disruptive child behavior is associated with low positive parenting and high negative parenting in this dataset (Wymbs, 2011). Confederate child behavior was negatively associated with positive parenting for mothers and fathers. Additionally for mothers and fathers, confederate child behavior was negatively associated with partner-rated negative, partner-rated positive, and observer-coded negative parenting, respectively.

### Moderation Analyses

#### Aim One

**Fig. 1:**
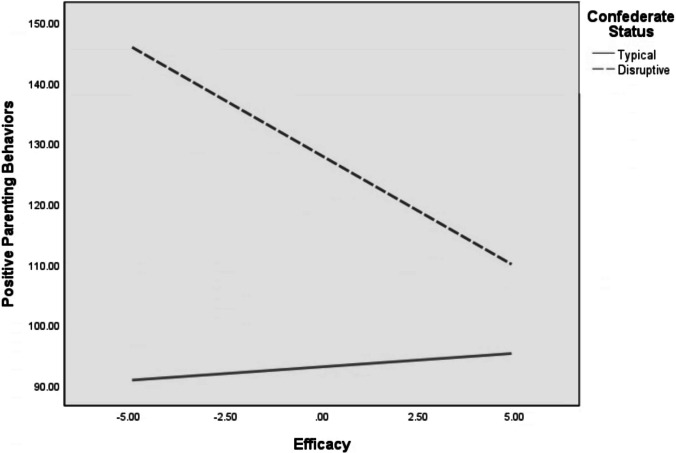
Moderating effects of mothers’ self-reported parenting efficacy on the association between confederate status and frequency of observer-coded positive parenting behaviors

Results of the regression analyses testing parenting self-esteem constructs (i.e., satisfaction and efficacy) as moderators of associations between confederate child behavior and parenting behavior outcomes with mothers are presented in Table 3. Interactions between confederate child behavior and each moderator significantly predicted observer-coded positive parenting. Examining the simple slopes of the interaction showed that when mothers interacted with a disruptive confederate and reported low levels of efficacy, the negative association between disruptive child behavior and positive parenting behaviors is significantly different from zero, and stronger than when mothers reported high levels of efficacy (Fig. 1). The Johnson-Neyman technique showed that the association between disruptive child behavior and positive parenting behavior was significant when self-reported efficacy was less than 2.785. This same pattern was also present for the interaction between confederate child behavior and satisfaction, such that mothers who interacted with disruptive confederates and reported low levels of satisfaction exhibited more frequent observer-coded positive parenting behaviors than mothers who interacted with disruptive confederates and reported high levels of satisfaction or mothers who interacted with typical confederates, regardless of satisfaction level (See Fig. 1 for pattern). The Johnson-Neyman technique showed that the association between disruptive child behavior and positive parenting behavior was significant when was self-reported satisfaction less than 3.787. However, no other interactions predicted parenting outcomes for mothers.

**Table 3:** Regression analyses predicting parenting outcomes for mothers and fathers

	OC Positive Parenting	OC Negative Parenting	PR Positive Parenting	PR Negative Parenting
Mother Education Level	6.47 (3.06)^*^	0.01 (0.02)	0.01 (0.08)	0.01 (0.32)
Length of Relationship w/ Child	-.11 (1.15)	0.00 (0.01)	0.03 (0.03)	-0.07 (0.09)
Confederate Status	34.87 (10.11)^**^	0.34 (0.36)	-1.70 (0.28)^**^	1.19 (0.28)^**^
*Efficacy*	-1.69 (1.04)	-0.00 (0.01)	0.06 (0.03)^*^	-0.03 (0.03)
Confederate Status x *Efficacy*	-4.10 (2.11)^*^	0.00 (0.01)	0.08 (0.06)	-0.08 (0.06)
Overall R^2^	**0.21**	**0.39**	**0.34**	**0.20**
Mother Education Level	8.01 (3.04)	0.02 (0.02)	0.02 (0.09)	-0.06 (0.09)
Length of Relationship w/ Child	0.75 (1.13)	0.00 (0.01)	0.02 (0.03)	0.02 (0.03)
Confederate Status	32.44 (9.99)	0.64 (0.35)^*^	-1.66 (.29)^**^	1.19 (0.28)^**^
*Satisfaction*	-1.73 (0.75)	0.00 (0.01)	.00 (.02)	0.00 (0.02)
Confederate Status x *Satisfaction*	-2.55 (1.49)^**^	-0.01 (0.01)	-0.04 (0.04)	0.00 (0.04)
Overall R^2^	**0.23**	**0.34**	**0.31**	**0.18**
Father Education Level	-0.27 (2.04)	-0.01 (0.02)	0.05 (0.07)	-0.07 (0.06)
Length of Relationship w/ Child	-1.71 (0.89)	-0.012 (0.01)	-0.01 (0.03)	-0.01 (0.03)
Confederate Status	22.73 (7.97)^**^	0.27 (.36)	-1.17 (.29)^**^	0.95 (0.22)^**^
*Efficacy*	2.39 (0.82)^**^	-0.00 (0.01)	0.09 (0.03)^**^	-0.06 (0.02)^**^
Confederate Status x *Efficacy*	3.14 (2.63)^*^	0.00 (0.01)	0.03 (0.06)	-0.05 (0.05)
Overall R^2^	**0.23**	**0.30**	**0.26**	**0.28**
Father Education Level	-0.66 (2.19)	-0.01 (0.02)	0.01 (0.07)	-0.06 (0.06)
Length of Relationship w/ Child	-1.08 (0.93)	-0.01 (0.01)	0.016 (0.03)	-0.02 (0.03)
Confederate Status	23.81 (8.38)^**^	-0.04 (.34)	-1.10 (0.28)^**^	0.92 (0.23)^**^
*Satisfaction*	1.14 (0.60)	-0.00 (0.01)	0.07 (0.02)^**^	-0.03 (0.02)
Confederate Status x *Satisfaction*	0.80 (1.22)	0.01 (0.01)	0.02 (0.04)	-0.01 (0.03)
Overall R^2^	**0.15**	**0.31**	**0.30**	**0.25**

*N* = 90

Unstandardized betas are presented in the table with the standard error following in parentheses

*PR* Partner ratings of parenting behavior, *OC* Observer coded parenting behavior

^*^p < 0.05; ^**^p < 0.01

**Fig. 2:**
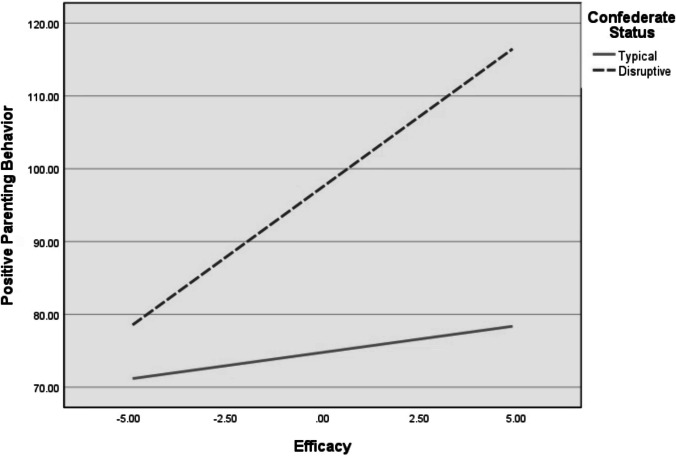
Moderating effects of fathers’ self-reported parenting efficacy on the association between confederate status and frequency of observer-coded positive parenting behaviors

Results of the same regression analyses for fathers are presented in Table 3. There was one significant interaction between confederate child behavior and parenting self-esteem (i.e., satisfaction and efficacy) in the prediction of parenting behavior. In contrast to results with mothers, fathers who interacted with the disruptive confederates and reported low levels of efficacy had a stronger negative association between disruptive child behaviors and positive parenting behaviors (Fig. 2). The Johnson-Neyman technique showed that the negative association between disruptive child behavior and positive parenting behavior was significant when self-reported efficacy is greater than 0. No other interactions predicted parenting outcomes for fathers.

### Exploratory Aims

**Fig. 3:**
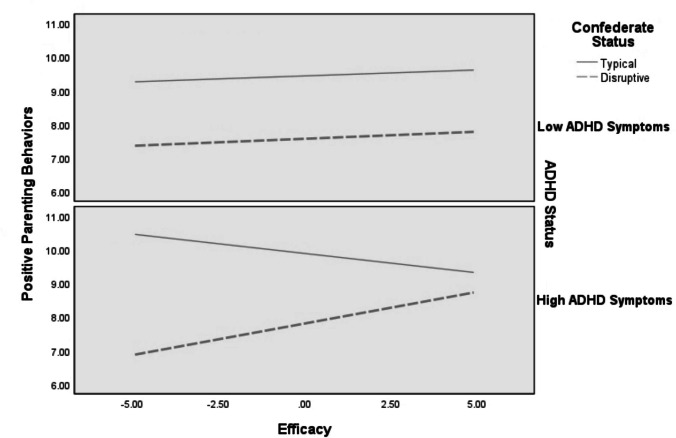
Moderating effects of mothers’ self-reported parenting efficacy and ADHD Status on the association between confederate status and partner-rated positive parenting behaviors

Linear regressions were used to test the three-way interaction between ADHD/ODD status of the parent couples’ own child, confederate child behavior, and parenting self-esteem domains (i.e., satisfaction and efficacy) as a predictor of parenting behavior outcomes for mothers (see Table 4). There was only one significant three-way interaction for parenting behavior outcomes for mothers (see Fig. 3): Mothers who interacted with a disruptive confederate, reported low efficacy, and had a child with ADHD had a stronger negative association between disruptive child behaviors and positive parenting behaviors than those who interacted with a typical confederate, or mothers who rated their efficacy at or one standard deviation above the mean, or who had a child without ADHD. Otherwise, the three-way interaction did not predict maternal parenting behavior. For fathers, there were no significant three-way interactions for parenting behavior outcomes (see Table 5), suggesting associations with parenting outcomes did not vary for fathers depending on their experience with raising an ADHD child or not.

**Table 4:** Exploratory analyses assessing three way interactions predicting parenting outcomes for mothers

	OC Positive	OC Negative	PR Positive	PR Negative
Mother Education Level	7.22 (3.21)	0.02 (0.02)	-0.01 (0.09)	-0.06 (0.09)
Length of Relationship w/ Child	0.36 (1.26)	0.01 (0.01)	0.02 (0.03)	0.03 (0.04)
Confederate Status	34.32 (11.52)^**^	0.11 (0.86)	-1.96 (0.31)^**^	1.26 (0.32)
*Efficacy*	-1.24 (1.26)	-0.00 (0.02)	0.04 (0.03)^*^	-0.02 (0.04)
Confederate Status x *Efficacy*	-4.01 (2.57)	0.01 (0.03)	0.14 (0.07)	-0.09 (0.07)
Child ADHD Status	12.54 (12.45)	-0.03 (0.72)	-0.33 (0.33)	0.27 (0.35)
Confederate Status x Child ADHD Status	1.87 (23.64)	0.25 (0.96)	0.22 (0.63)	0.07 (0.66)
Child ADHD Status x *Efficacy*	-0.36 (2.66)	0.00 (0.02)	-0.00 (0.07)	-0.01 (0.07)
Confederate Status x Child ADHD Status x *Efficacy*	-0.94 (5.36)	-0.01 (0.03)	-0.30 (0.14)^*^	0.07 (0.15)
Overall R^2^	**0.22**	**0.40**	**0.38**	**0.21**
Mother Education Level	7.97 (3.10)^*^	0.02 (0.02)	-0.01 (0.09)	-0.04 (0.09)
Length of Relationship w/ Child	1.13 (1.17)	0.00 (0.00)	0.01 (.03)	0.03 (0.04)
Confederate Status	30.60 (10.78)^**^	0.83 (0.73)	-1.88 (0.31)^**^	1.21 (0.32)^**^
*Satisfaction*	-0.93 (0.82)	-0.00 (0.01)	0.00 (0.024)	0.01 (0.02)
Confederate Status x *Satisfaction*	-1.67 (1.66)	-0.01 (0.02)	-0.03 (0.048)	0.01 (0.05)
Child ADHD Status	13.15 (11.64)	-0.05 (0.60)	-0.47 (0.34)	0.32 (0.34)
Confederate Status x Child ADHD Status	2.52 (21.85)	-0.23 (0.86)	-0.33 (0.63)	0.39 (0.65)
Child ADHD Status x *Satisfaction*	-4.045 (1.72)^*^	0.00 (0.04)	-0.00 (0.05)	0.01 (0.05)
Confederate Status x Child ADHD Status x *Satisfaction*	-4.39 (3.40)	0.01 (0.02)	-0.15 (0.10)	0.01 (0.10)
Overall R^2^	**0.30**	**0.40**	**0.35**	**0.19**

*N* = 90

Unstandardized betas are presented in the table with the standard error following in parentheses

*PR* Partner ratings of parenting behavior, *OC* Observer coded parenting behavior

^*^p < 0.05; ^**^p < 0.01

**Table 5:** Exploratory analyses assessing three way interactions predicting parenting outcomes for fathers

	OC Positive	OC Negative	PR Positive	PR Negative
Father Education Level	0.04 (2.14)	-0.01 (0.02)	0.01 (0.08)	-0.05 (0.06)
Length of Relationship w/ Child	-1.07 (0.94)	0.01 (0.01)	-0.03 (0.03)	0.01 (0.03)
Confederate Status	27.01 (8.72)^**^	-0.13 (0.77)	-1.20 (0.31)^**^	0.90 (0.24)^**^
*Efficacy*	2.31 (0.92)^*^	-0.00 (0.02)	0.06 (0.03)	-0.04 (0.03)
Confederate Status x *Efficacy*	2.54 (1.84)	0.01 (0.03)	-0.00 (0.07)	-0.02 (0.05)
Child ADHD Status	1.55 (9.14)	0.01 (0.67)	-0.63 (0.33)^*^	0.35 (0.25)
Confederate Status x Child ADHD Status	-4.34 (17.35)	0.42 (0.89)	-0.83 (0.62)	0.56 (0.48)
Child ADHD Status x *Efficacy*	1.42 (1.93)	-0.00 (0.02)	0.06 (0.07)	-0.07 (0.05)
Confederate Status x Child ADHD Status x *Efficacy*	4.42 (3.90)	-0.01 (0.03)	-0.01 (0.14)	-0.05 (0.11)
Overall R^2^	**0.25**	**0.31**	**0.31**	**0.32**
Father Education Level	-0.52 (2.25)	-0.01 (0.02)	0.00 (0.08)	-0.04 (0.06)
Length of Relationship w/ Child	-0.75 (1.07)	-0.01 (0.01)	0.01 (0.04)	-0.01 (0.03)
Confederate Status	29.50 (10.07)^**^	-0.39 (0.74)	-1.07 (0.34)^**^	1.07 (0.27)^**^
*Satisfaction*	1.27 (0.75)	-0.00 (0.01)	0.06 (0.03)^**^	-0.02 (0.02)
Confederate Status x *Satisfaction*	0.06 (1.47)	0.02 (0.02)	-0.01 (0.05)	0.01 (0.04)
Child ADHD Status	4.63 (10.90)	0.14 (0.68)	-.246 (0.37)	0.31 (0.30)
Confederate Status x Child ADHD Status	-12.81 (20.14)	0.20 (0.85)	-0.867 (0.68)	0.57 (0.55)
Child ADHD Status x *Satisfaction*	0.91 (1.50)	-0.00 (0.02)	0.01 (0.05)	-0.01 (0.04)
Confederate Status x Child ADHD Status x *Satisfaction*	2.43 (3.01)	0.00 (0.02)	0.02 (0.10)	0.07 (0.08)
Overall R^2^	**0.17**	**0.34**	**0.32**	**0.28**

*N* = 90

Unstandardized betas are presented in the table with the standard error following in parentheses

*PR* Partner ratings of parenting behavior, *OC* Observer coded parenting behavior

^*^p < 0.05; ^**^p < 0.01

## Discussion

The effects of children with disruptive behaviors on parenting behavior span decades of research. Prospective and experimental manipulation studies have demonstrated that disruptive child behaviors increase risk of parents engaging in more negative and less positive parenting (Breaux & Harvey, 2019; Pelham et al., 1998). However, it is unlikely that all parents are at the same degree of risk associated with managing disruptive child behavior. Further investigation examining the degree to which parents with certain risk factors may be more susceptible to responding more negatively and less positively in the moment to disruptive child behavior is warranted, as this work could inform who to target for interventions and potentially how to help them. The results of this study highlight that the associations between disruptive child behavior and parenting behavior did not often vary in a meaningful way for mothers or fathers with high or low parenting self-esteem constructs (i.e., satisfaction and efficacy). Although patterns in significant findings were often consistent between mothers and fathers, more often than not findings did not align with proposed hypotheses, especially when examining negative parenting behaviors. Additionally, significant results were only found when utilizing observer-coded parenting behaviors, thus implying that the association between disruptive child behaviors and partner-rated parenting behaviors do not vary meaningfully by satisfaction or efficacy.

For the first aim, the study investigated whether links between disruptive child behavior and negative and positive parenting behaviors were stronger or weaker depending on the level of parenting self-esteem endorsed. Surprisingly, mothers reporting low parenting satisfaction and efficacy, which the literature indicates portends greater risk of more negative and less positive parenting (Johnston, 1996; Johnston & Mash, 1989; Ohan et al., 2000), responded better to disruptive child behaviors in the moment (with more positive parenting) than mothers endorsing higher parenting satisfaction and efficacy. These findings seem to be inconsistent with past research, such that mothers’ positive parenting behaviors were not found to be positively associated with self-reported efficacy and satisfaction scores as in prior investigations (Ohan et al., 2000). A reason for this could be that mothers feel a sense of helplessness with their own child but feel more confident in their abilities to manage the behavior of an unfamiliar child engaging in disruptive behaviors. Another possibility for this may be that mothers with low parenting self-esteem (i.e., satisfaction and efficacy) may be overly engaging in positive behaviors during the interaction due to feel less confident in their parenting abilities, as well as interacting with an unfamiliar child and being observed. These inconsistencies may be due to previous research using self-reported, typical parenting behaviors instead of those relied on during in the moment management of disruptive child behaviors. Self-report measures of parenting behavior usually assess the parent’s perception of their average parenting behavior across time and often miss unique behaviors observed while managing disruptive child behavior. That said, this interaction between a parent and an unfamiliar child may have influenced these results, as well as parents knowing they were being observed during the interaction. Thus, a potential direction for researchers is to have parents interact with their own child and manipulate child behavior through use of a medication manipulation.

In contrast to findings with mothers, and aligned with the original hypothesis, the positive association between disruptive child behavior and positive parenting was stronger for fathers who reported high parenting efficacy scores than fathers reporting low efficacy scores. That is, fathers who endorsed low levels of parenting efficacy were less likely to engage in positive parenting behaviors in the moment when managing disruptive behaviors compared to fathers reporting high levels of parenting efficacy. As expected, based on prior research (Ohan et al., 2000), fathers who feel less efficacious in their roles tend to respond less positively to disruptive child behaviors in the moment. This pattern may be apparent for fathers and not for mothers due to fathers’ sense of satisfaction and efficacy being more sensitive to child misbehavior compared to mothers due to caretaking role diffusion that occurs between parents. Past research examining parenting self-esteems speculates that fathers may not build resilience to disruptive child behaviors due to not taking on the primary caretaking role and engaging in more coercive interactions with their child compared to mothers (DeGarmo et al., 2016). Future research should look to test for differences in patterns between mothers and fathers as well as potential mechanisms for differences in those interactions, such as diffusion of parenting responsibility.

Regarding exploratory analyses with parenting outcomes, the association between mothers who interacted with the disruptive confederate, reported low efficacy, and had a child with ADHD were likely to be rated by their partner as engaging in less positive parenting behaviors than mothers who reported high levels of efficacy. In sum, it appears that during these triadic interactions mothers who had a child with ADHD, reported low efficacy, and engaged with a disruptive confederate were perceived by their partner to have engaged in less positive parenting behaviors compared to mothers who had a child with ADHD, reported high levels of efficacy, and engaged with a disruptive confederate. This finding is in contrast to our earlier finding that showed mothers who endorsed low parenting efficacy showed the strongest association between disruptive child behavior and positive parenting behavior. Thus, it is not only parenting efficacy that may be important, but also child ADHD/ODD status may be a key factor in changing the associations between disruptive child behavior and parenting behaviors as research has shown that parents of children with disruptive behavior disorders (i.e., ADHD) tend to engage in more negative parenting behaviors compared to parents of children without ADHD (Johnston & Chronis-Tuscano, 2015). Additionally, it may be that who is rating the parenting behavior may be important in these result patterns as partner’s perception of parenting behaviors were not significant for mother in the previous moderation analyses. Thus, partner’s perceptions of behavior may also be an important factor in the differences between the previous primary analyses and the exploratory ones. The current analyses controlled for years of parenting experience, so ADHD/ODD status of their own child may indicate depth of experience mothers have with raising children with difficult behaviors and that mothers may be more sensitized to these behaviors in regard to their positive parenting. Due to this same pattern not being observed with fathers and no other parenting outcomes were predicted by this interaction, it is important to not overly interpret the role of parenting self-esteem in this process with moms. If replicated, these findings may provide a reason to engage mothers in parent training interventions that also serve to bolster believed efficacy by equipping parents with behavioral management strategies to prevent engagement in less positive parenting behaviors when managing disruptive child behaviors.

### Limitations and Future Directions

Although this study has many strengths, such as utilizing an experimental manipulation study design and observer-coded parenting behaviors and analyzing patterns separately for mothers and fathers in the context of co-parenting interaction, interpreting the results of this study needs to occur with several limitations in mind. First, though many of the findings presented in this study align with past research examining parents and their own children (and thereby disentangling any potential shared genetic variance), the chance that interacting with an unknown child outside of the context of their home triggered response bias resulting in unusual parenting behavior. These findings may reflect under-estimates of negative parenting behaviors, as parents may be interacting more positively with the confederate during the interaction than they would their own child. However, many studies using confederate children have still found robust effects despite parents interacting with an unfamiliar child (Brunk & Henggeler, 1984; Bugental et al., 1980; Pelham & Lang, 1999; Wymbs & Pelham, 2010). Second, the interaction occurred in the context of a triad, which included two parents interacting with the child. One parents behavior, communication, and change in affect may be influenced by the other parents’ presence, which violates the underlying assumption of case independence for linear regression analyses. Third, the current study utilized a sample that was predominantly white, middle- to upper-class, married, and parents of 9- to 12-year-old boys. Previous research indicates that socio-economic disadvantage such as low parent education or family income, is linked with poorer parenting (Reyno & McGrath, 2006). Additionally, single parents and parents from underserved backgrounds have often reported differences in parenting behaviors and stress, which are associated with parenting self-esteem levels (Chung et al., 2024; Kazdin et al., 1997; Robinson & Harris, 2013), which affect the generalizability of the results of this study. With this research in mind, parents who participated in the study may have meaningfully different parenting behaviors compared to a more diverse set of parents (i.e., single parents, SES, race/ethnicity). Parenting behavior and characteristics (i.e., warmth, responsiveness) have also shown to change over the child development spectrum to meet the needs of the child and promote autonomy, thus it would be important to consider how parent behavior differs for children that are younger or older than the samples (Mowder, 2005).

With both the findings and limitations in mind, there are many potential directions for continued research that both extends and complements the current study. First, despite the lack of evidence underscoring parenting self-esteem as a moderator in this study, it remains to be that risk of negative responses to disruptive child behavior may be greater in some parents than others. There remains a need to evaluate alternative risk factors that may have moderating effects on the association between disruptive child behaviors. Additional factors could include feelings of satisfaction within the interparental relationship, perceptions of partner’s efficacy and satisfaction, and mental health concerns (i.e., ADHD, depression). Parents reporting mental health concerns have been shown to have lower ratings of parenting efficacy and satisfaction (Fang et al., 2021), as well as show different patterns in parenting behaviors compared to parents without mental health concerns (Wymbs et al., 2015). Previous research has identified a trend for parenting partner’s efficacy to be positively associated with one another, as well as have associations with parenting behaviors when managing disruptive child behavior (Margolin et al., 2001). Additionally, parents reporting low marital satisfaction often have children with disruptive behaviors, often report low parenting efficacy, and demonstrate more negative parenting behaviors (Johnston & Mash, 1989; Jouriles et al., 1988). Notably, preliminary analyses using the current dataset specifically examine moderating effects of parenting efficacy and parental mental health have shown that associations between disruptive child behavior and parenting outcomes may indeed vary in strength depending on parent depressive and ADHD symptoms (Hightower et al., 2023). Another potential factor to consider for future research is different levels of disruptive child behaviors (e.g., ADHD only vs. ADHD and comorbid ODD). Previous research highlights differences in levels of satisfaction and efficacy when comparing parents of children diagnosed with ADHD to parents of children diagnosed with ADHD and ODD (Johnston, 1996).

Second, another potential line of work that could further this area of research is examining potential mechanisms (e.g., negative affect, parent psychopathology, parental stress) that account for the relation between managing disruptive behaviors and negative parent outcomes (i.e., interparental discord and negative parenting behaviors) for parents who report low parenting self-esteem. Prior work has highlighted that parents who have a child with chronic disruptive behaviors and low parenting self-esteem report greater parenting stress, greater symptoms of psychopathology, and are more susceptible to experiencing greater negative affect compared to parents of children without ADHD and high parenting self-esteem (Chronis et al., 2003; Johnston et al., 2012). Thus, these may be potential mechanisms that drive the relationships between disruptive child behavior and negative parenting behaviors in parents of children with ADHD who report low parenting self-esteem.

### Conclusions

The current study provides evidence that indicates parenting self-esteem serves as a moderator on the association between child behavior and positive parenting behavior. At the same time, the current study suggests that parenting self-esteem is not a consistent moderator of the relationship between disruptive child behaviors and negative parenting behaviors. Thus, research remains needed to examine how parenting cognitions interact with disruptive child behaviors to influence negative parenting and other relevant outcomes like communication. Disruptive child behaviors may lead to this skewed sense of satisfaction and efficacy that may prevent the use of positive parenting behaviors when responding to disruptive behaviors in the moment, which is why it may be important to address parenting self-esteem in the context of working with parents. If findings are replicated, this may indicate a need to address maladaptive parental cognitions impacting satisfaction and efficacy in parents of children with ADHD before beginning behavioral parent training interventions as their sense of parenting self-esteem may hinder the use of effective strategies when responding to in the moment disruptive behaviors from their child. Parental cognitions like parenting self-esteem impact the way parents think about their role as parents, thus influencing how they interact with their child. By addressing negative cognitions regarding their parental abilities may lead to increased engagement in behavioral parent training sessions and later adoption of strategies learned in sessions.

## Data Availability

The data generated during and/or analyzed during the current study are available on reasonable request.
